# Nutritional Interventions in Children with Brain Injuries: A Systematic Review

**DOI:** 10.3390/nu13041130

**Published:** 2021-03-30

**Authors:** Tamy Colonetti, Maria Laura R. Uggioni, Sarah D. Ferraz, Marina C. Rocha, Mateus V. Cruz, Maria Inês da Rosa, Antonio J. Grande

**Affiliations:** 1 Laboratory of Biomedicine Translational, Postgraduate Program in Health Sciences, Universidade do Extremo Sul Catarinense (UNESC), Criciúma 88806000, SC, Brazil; lala@unesc.net (M.L.R.U.); sarahdferraz@gmail.com (S.D.F.); marina_costa98@hotmail.com (M.C.R.); mateus_cruz11@hotmail.com (M.V.C.); mir@unesc.net (M.I.d.R.); 2 Laboratory of Evidence-Based Practice, Universidade Estadual de Mato Grosso do Sul, Campo Grande 79804970, MS, Brazil; grandeto@gmail.com

**Keywords:** brain injuries, nutrition therapy, child

## Abstract

Background: Brain injury has several consequences throughout life, its increased incidence has caused great public concern. The aim was identifying the main nutritional therapies recommended for children with brain injuries. Methods: A systematic review was carried out using the terms in the search strategy: “Brain Injuries”, “Nutrition Therapy”, and “Child” and their synonyms, from database inception up to August 2020. The search was conducted in the following databases: MEDLINE, EMBASE, Scopus, Cochrane library, LILACS, and grey literature. Two reviewers independently selected the included studies, according to the eligibility criteria and extracted data from the included articles. Results: A total of 1196 studies resulted from electronic searches, and out of these, 27 studies were read in full and eight studies were included. For early enteral nutritional support (<48 h), results suggest benefit in increasing survival rates. Type of dietary protein seems to be related to decreasing gastric emptying by 40%. The use of fibers seems to reduce gastrointestinal discomfort and increase stool frequency. Conclusions: The evidence mapped was extracted from small studies analyzing different outcomes, so any decision making should be analyzed considering the context. We present the direction of the effect, but the magnitude is still unclear.

## 1. Introduction

Traumatic brain injury (TBI) is characterized by a dysfunction in the brain which could potentially causes a cognitive deficit related to attention, language, and memory, behavioral changes and physical deficiencies causing loss of motor control and balance, directly affecting the performance of daily activities. TBI in infants has worse prognosis compared to adults as the brain of a child is still developing. Disorders can occur in work memory, planning, inhibition, decision making, and social interactions [1].

Although brain injury has several consequences throughout life, its increased incidence has caused great public concern. In the world, it is estimated that more than 10 million brain injuries occur every year. Moderate to severe injuries can either lead to hospitalization or end up in death [2]. Between the years 2005 and 2009, approximately 2 million children had outpatient consultations and nearly 3 million children were admitted to an emergency room with mild TBI in the world [3].

Cerebral palsy (CP) is the result of a brain injury that occurs before the brain is fully developed, that is, before the age of two, which means that this injury can occur in the prenatal, perinatal, or post-natal period and is a permanent neurological disorder [4]. Thus, non-progressive syndromes are characterized by decreased posture and movements, resulting in the central nervous system malformation. CP is usually caused by brain injuries in the first months or years of life, being linked to several factors, such as: genetic factors, predisposition to infections, trauma, and acute asphyxia both during pregnancy and at the time of delivery [5].

The prevalence (1.8 to 5.9 per 1000 live births) and severity of CP make it a public health problem [6]. Worldwide, the incidence of CP is estimated at two per 1000 live births [7]. The lifetime cost for supporting children with CP is estimated at approximately US $ 1 million dollars considering expenses with health, education, and social services [6].

Children diagnosed with CP are at high risk of developing nutritional deficiencies, hindering their growth, as these children commonly have eating and swallowing disorders, which increases the risk of aspiration when fed orally, in addition, they may still have short stature and weights below what is considered healthy for their age [8,9].

There are multiple factors that trigger nutritional deficiencies, the main ones being: oropharyngeal dysphagia, reflux, occlusal changes, behavioral disorders, and communication difficulties [10]. The consequences of this nutritional status can lead to impaired brain function and reduced immune response—increasing the risk of infections and decreasing respiratory muscle strength. These complications can cause a worse quality of life and decrease life expectancy [10].

Therefore, the objective of this paper is to identify the main nutritional therapies recommended for children with brain injuries.

## 2. Materials and Methods

This is a systematic review synthesizing scientific evidence on the nutritional therapies recommended for children with brain injuries. This study was registered at PROSPERO (International Prospective Register of Systemic Reviews, http://www.crd.york.ac.uk/prospero; accessed on 21 August 2020, CRD42020200211). The review was performed according to a prospective protocol using PRISMA (Preferred reporting items for systematic reviews and meta-analyses). 

### 2.1. Eligibility Criteria

For selection criteria, randomized clinical trials on nutritional interventions performed with children with CP or TBI compared to a control group were included.

P: Children with brain injury (CP or TBI)

I: Nutritional interventions

C: Control group (Children with brain injury without intervention)

O: Therapies implemented (enteral route of food administration; oral and enteral nutrition; oral nutrition only). Results found with the intervention (improvement of gastrointestinal symptoms such as reflux, constipation, gastric emptying, weight gain, neurological growth, and development).

S: Randomized Controlled Trials 

### 2.2. Information Sources and Search Strategy

The search was conducted in the following electronic databases: MEDLINE (PubMed), BVS (LILACS and others), Embase (Elsevier), and The Cochrane Library. The databases were searched using the following terms: “Brain Injuries”, “Nutrition”, “Child”” and their synonyms, from database inception up to August 2020. Searches were limited to humans, regardless of the language published. The reference lists of all selected studies were explored for relevant studies, also grey literature. 

### 2.3. Study Selection

Two review authors (TC and MLU) independently assessed the titles and abstracts of all articles identified by the searches. Once the authors excluded the studies which did not meet the criteria, the remaining studies were read in full. A third review author (MIR) resolved any disagreements in selection of included studies. The screening process was conducted at Rayyan (rayyan.qcri.org (accessed on 21 March 2020)). 

### 2.4. Data Extraction

Two researchers (MC and SDF) independently extracted data on participants, interventions, and outcomes, as described above in the selection criteria section using a standardized form. 

The data extraction form was composed by author, year, country, patient characteristics in each group, methods, intervention, and placebo information and results of each included study.

### 2.5. Quality Assessment

All included studies were evaluated for their methodological quality through the Cochrane Collaboration tool for assessing the Risk of Bias (RoB 2). The Risk of Bias domains consists of bias resulting from the randomization process; deviations from the intended intervention; missing outcome data; measurement of the outcome; and selection of reported result. For each assessed outcome, we used the GRADE approach to assess the quality of the evidence produced by this systematic review.

### 2.6. Data Synthesis and Analysis

To perform a quantitative assessment of interventions, at least two of the included studies should present similar interventions in terms of their methodological characteristics and measurement and presentation of outcomes. 

Meta-analysis was not performed due to the different interventions evaluated in the studies included in this review, that is, clinically, these therapies should not be mixed. Thus, a descriptive synthesis of the studies was carried out. The results were presented in tables.

## 3. Results

### 3.1. Selection of Included Studies

**Figure 1:**
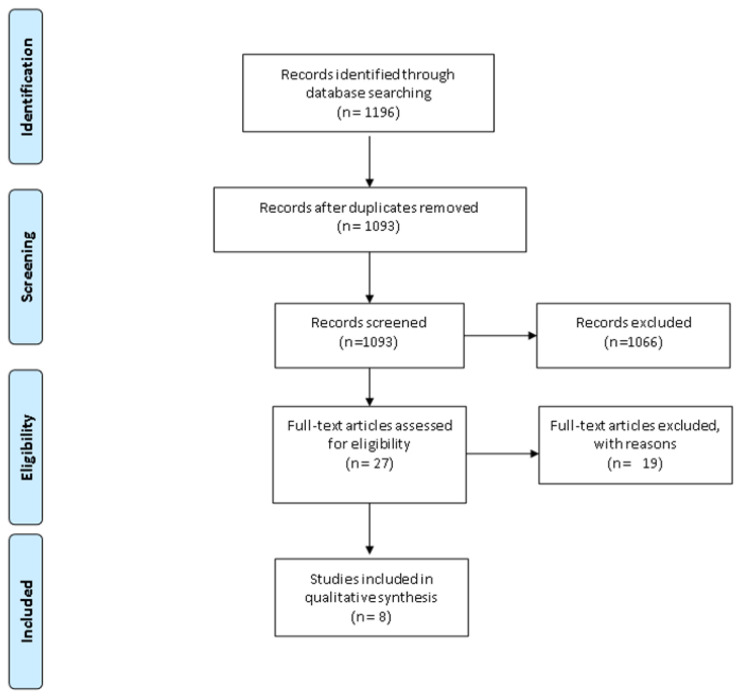
Flow diagram of information through the different phases of a systematic re-view.

After a search strategy in the databases, 1196 studies were found, of which 103 were duplicates and therefore, excluded, leaving 1093 studies for titles and abstracts screening. Out of these, 1066 were excluded because they did not qualify for full-text reading, leaving 27 studies. Out of the 27 studies selected for full-text reading, 19 studies were excluded, four because they are study projects/protocols already included in their final published version or study projects with no results and 15 because they are observational studies. Resulting in eight included studies for this review [11,12,13,14,15,16,17,18], with six studies exploring CP and two of them exploring TBI in children (Figure 1).

### 3.2. Characteristics of Included Studies

This systematic review included studies published between 2000 and 2018, including the following countries: Japan [11], Italy [12], United States of America [13,18], United Kingdom [14,16], Australia [15], and Greece [17]. Most of the studies were carried out in hospitals or children’s centers. In total, the eight RCTs included in this review presented 289 participants. The age of the study participants ranged from 1 month to 15 years old, including female and male children, with a larger male population. Considering nutrition, five studies [11,13,15,17,18] presented enteral route of food administration, one study [16] presented oral and enteral, and two other studies [12,14] presented only oral nutrition. The interventions sought to evaluate results in the improvement of gastrointestinal symptoms such as reflux, constipation, gastric emptying, weight gain, neurological growth, and development. The characteristics of the included studies are shown in Table 1.

### 3.3. Risk of Bias Assessment

**Figure 2:**
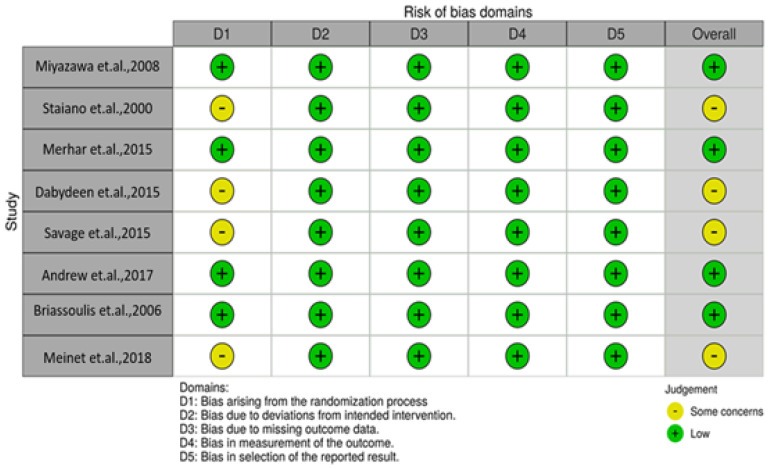
Risk of Bias of randomized clinical trials included in the study.

The risk of bias assessment was performed using the Cochrane Collaboration tool for assessing the Risk of Bias (RoB 2.0) and is shown in Figure 2.

Regarding randomization process (Domain 1): The studies [12,14,15,18] reported being randomized, but they did not explain how the randomization and allocation of patients was carried out, being classified as “Some concerns”—some doubts regarding this stage of the research. The other studies [11,13,16,17] were classified as low risk of bias because they described the randomization process in detail. Deviations from the intended intervention (Domain 2): all included studies described how the intervention was prescribed in detail, including blinding, as a result, the domain was classified as low risk of bias. Missing outcome data (Domain 3): all included studies described the results from the participants randomized, including data on research objectives, as a result the domain was classified as low risk of bias. Measurement of outcome (Domain 4): all included studies used adequate methods for measuring the outcomes and the assessments did not differ from experimental and control groups [11,12,13,14,15,16,17,18], as a result the domain was classified as low risk of bias. Selection of the reported result (Domain 5): all included studies were registered at ClinicalTrials.gov. Thus, the information at registration and at publication were cross-checked, as a result, the domain was classified as low risk of bias. Overall bias: all the included studies was classified as low risk of bias.

### 3.4. Description of Nutritional Interventions

The nutritional interventions vary widely, but most seek to improve gastrointestinal discomfort and assist in the growth and development of these children (Table 2). Two studies evaluated the use of additional fibers in the diet as pectin for GERD [11] and glucomannan for constipation [12]. One study increased daily protein intake to 4 g/kg for weight gain for EN in patients with TBI [13]. One study increased the energy intake and combined it with protein for body growth rate [14]. One study showed that increased daily protein intake for the improvement of GI discomforts in patients with EN [15]. One study used omega-3 associated with a combination of micronutrients for the improvement of neurological development [16]. One study assessed the supplementation with glutamine, arginine, antioxidants, and omega-3 fatty acids in the inflammatory process [17] and reported the findings for Glasgow Coma Scale, food intake, and mortality [18]. A detailed summary with the description of the nutritional interventions and the outcomes assessed for the eight included studies are shown in Table 2.

### 3.5. Quality of the Evidence

We assessed the certainty and strength of the evidence using the GRADE approach. As we have a systematic review without metanalysis, we descriptively assessed each outcome. The certainty of evidence for the following outcomes assessed were moderate (downgraded −1), the risk of bias was considered serious due to lack of crucial information on the methodology: (1−) glucomannan use for the treatment of chronic constipation, (2−) pectin use “% time pH < 4 in the upper and lower esophagus”, (3−) energy and protein intake in growth. For the outcome gastroesophageal reflux using whey protein-based enteral formulas, we found low quality evidence (downgraded −2), the risk of bias was considered serious (−1) with wide confidence interval (−1). For the outcome child growth, protein supplementation in the enterally route is safe and well tolerated, we found high quality evidence.

## 4. Discussion

This systematic review sought to identify the nutritional therapies studied for children with brain injury. Randomized controlled trials that evaluated children with CP or TBI were included. The nutritional interventions studied varied in terms of the types of nutrients and outcomes evaluated, but most of them aimed at improving gastrointestinal discomfort and assessing the growth and development of these children. Due to differences between the interventions, a meta-analysis was not possible to be carried out. 

The included studies showed that in order to prevent malnutrition, there should be a higher caloric-protein intake in children, and that the type of protein used has an influence on gastric emptying. Fiber intake seems to be important, since it is related to the improvement of gastrointestinal symptoms, but dose data are still lacking. There is still little literature on the adequacy and quality of dietary lipids, as well as the specific needs for micronutrients.

One of the studies included in this review [18] found a positive association between early onset of food introduction (<48 h) and survival, with reduced mortality rates in children with TBI. One study [19] carried out explored the effect of early enteral nutrition (EN) support (started within 48 h post trauma) on the survival rate, using the Glasgow Coma Scale (GCS) score and assessed the clinical outcome of patients with TBI. During the years 2002–2010, data from 145 patients with early EN who received appropriate calories and nutrients within 48 h post trauma were collected and compared with 152 non-early EN controls, matched for sex, age, body weight, GCS initial score, and operative status. Patients with early EN presented higher survival and GCS score on the 7th day of admission to the intensive care unit and better prognosis 1 month after the injury. Non-early EN patients had a risk ratio of 14.63 (95% CI 8.58–24.91) compared with early EN patients. The GCS score during the first 7 days of the ICU was significantly improved among early EN patients with GCS scores of 6–8. These findings demonstrate that EN within 48 h after the injury is associated with better survival.

Another study [20] explored the current practice of EN in children with TBI and the risk factors associated with late onset of EN by classifying participants into two groups (early (≤48 h) and late (>48 h). In total, 416 patients from the study were included in five pediatric trauma centers, with 347 patients in the Early EN group, with a mean age of 4.24 years and 69 participants in the Late EN with a mean age of 6 years. The overall mortality was 2.6%. Delayed EN was independently associated with worse functional status at discharge from the ICU (*p* = 0.02), but it was not associated with mortality or increased length of hospital stay. Another study [21] analyzed the moment of beginning the nutritional supplement and the moment of reaching total caloric intake in relation to the length of hospital stay (LOS) in the intensive care unit. A total of 109 children between 8 and 18 years old with TBI were evaluated. The average time to start nutrition was 1.5 days (0.02–11.9 days), and the total caloric objective were reached in 3.4 days (0.5–19.6 days). The average length of stay in the ICU was 2.1 days (0.01–97.9 days). Overall, 48% of patients were discharged; 28% experienced mild, moderate, or severe disability; and 24% died or survived in a vegetative state. The early onset and the reach of total caloric intake were both positively correlated with shorter ICU stay (*p* < 0.01) and better mood at discharge (*p* < 0.05).

Three studies [13,14,17] assessed different nutritional interventions to weight gain and growth in children with CP and TBI. One study [22] assessed the nutritional status of children with CP in Vietnam, including participants from 0 to 18 years old who attended the National Children’s Hospital Hanoi. A total of 765 children with an average age of 2.6 ± 2.5 years were analyzed, of which 28.9% (*n* = 213) were underweight and 29.0% (*n* = 214) had rickets. The chances of underweight were significantly higher among children aged >5 years and /or with a monthly family income <50 USD. Low weight and/or short height was higher among children with quadriplegia (81%, *n* = 60 and 84.5%, *n* = 87), Gross Motor Functional Classification System (GMFCS) level IV–V (62.5 %, *n* = 45 and 67.0%, *n* = 67). Almost a third of children with intellectual disabilities and more than half of children with hearing impairments were underweight and/or short. For the authors in children with CP, the low economic level and the increase in motor severity increase the vulnerability to malnutrition.

One study [23] investigated the micronutrient balance in children with CP, aged 4 to 12 years fed orally (*n* = 12) or enteral (*n* = 9), compared to a control group of 16 children with healthy development. Parents collected data in duplicates of all the food their children ate for 3 consecutive days. Regarding children with CP fed orally, anthropometric data show significantly reduced z scores, weight = −1.92 (±1.59), height = −1.68 (±1.0), and BMI = −1.06 (±1.62) compared to controls. The enterally fed group, on the other hand, showed only reductions in z scores for height = −1.75 (±0.90) and weight = −0.73 (±1.24), but not BMI = 0.52 (±1.01). This was due to the lower z scores of weights in children fed orally, which also reached statistical significance when compared to children fed enterally. Protein intake was significantly higher in the control group compared to children fed orally and enterally (*p* = 0.005). Magnesium intake was significantly reduced in children with CP fed orally compared to the other groups. It was below the recommendations in 7 out of 12 of these children. Children with CP also had inadequate intake of calcium, copper, potassium, iodine, and manganese.

One study [15] evaluated the use of enteral formula based on whey versus casein in gastroesophageal reflux and gastric emptying in children with CP. For gastroesophageal reflux, there was no difference between the formulas, but gastric emptying was faster in the group that received whey-based formula. This result was similar to another study that evaluated the influence of protein composition on the rate of gastric emptying [24]. In total, 115 children with CP, who were using gastrostomy, participated in the study. The children received four meals on alternate days containing a carbohydrate and a fat base plus one of the four protein modules (100% casein, hydrolyzed whey, amino acids or 40% casein/60% whey) with a total energy of 1 kcal/mL. The gastric emptying time was slower in the meal with 100% casein and faster in the meal with 40% casein and 60% whey. Thus, CP children in gastrostomy, type of protein of the meal influences the gastric emptying.

Six studies [11,13,15,16,17,18] explored the type of feeding via EN. One study [25] evaluated the food supply through nutritional support, performed through nasoenteral tube or gastrostomy, on nutritional status and body composition in severely malnourished children with CP and spastic quadriplegia. In total, thirteen participants received food via a nasoenteral tube or gastrostomy. During the first two weeks, energy intake was 112 kcal/kg/d and in the following two weeks from 115 to 116 kcal/kg/d. From the sixth day onwards, iron was administered in daily doses of 3 mg/kg/d. During the fourth week of the study, significant increases were observed in anthropometric indicators, BMI, and weight/length (*p* < 0.01). The authors concluded that intensive nutritional support for four weeks had a significant effect on the nutritional status and body composition of malnourished children with CP and spastic quadriplegia, being an important strategy aimed at preventing malnutrition.

The literature is scarce on the topic, different interventions are analyzed, making it difficult for professionals to make evidence-based decisions. Thus, more research with interventions such as those included in this review, investigated by RCTs, are still necessary to improve nutritional inputs for the proper growth of children with brain damage.

## 5. Conclusions

The evidence mapped comes from small studies analyzing different outcomes, so any decision making should be analyzed considering the context. We present the direction of the effect, but the magnitude is still unclear. The literature on the subject suggests that early EN support (<48 h) increase survival rate in children with TBI [18]. In children with CP, the type of dietary protein seems to be related to gastric emptying [15] and the use of fibers seems to improve gastrointestinal discomfort [11,12], greater energy intake was observed in the first few years of life [4]. New RCTs must be conducted to answer what are the best options in nutritional therapy.

## Figures and Tables

**Table 1:** Characteristics of the studies included.

Author/Year	Country	Study Location	Total Population	Intervention Population	Gender	Age	Feeding Type	Aim of the Study
Andrew et al., 2017 [16]	United Kingdom	Children’s Center	32	14	28 M/12 F	1–18 months	Oral or enteral	The aim of the study was to investigate whether nutritional intervention with supplementation of DHA, choline, and uridine at the maximum permitted levels improves the results of neurological development in infants with suspected CP.
Briassoulis et al., 2006 [17]	Greece	Hospital- Pediatric intensive care unit	40	20	Uninformed	Average: 112 (months)	Enteral	Enteral feeding supplemented with glutamine, arginine, antioxidants, and omega-3 fatty acids in order to assess nutritional and metabolic indices and survival, length of stay, time of ventilation.
Dabydeen. et al., 2015 [14]	United Kingdom	Uninformed	16	8	9 M 7 F	12 months	Oral	Follow-up study 12 months after acute perinatal brain injury with a high-calorie and high-protein diet, in weight and height.
Meinert et al., 2018 [18]	USA	Uninformed	90	85 divided into 3 groups: Group 2 = 32; Group 3 *n* = 36; Group 4—*n* = 17)	Uninformed	Uninformed	Enteral	The authors sought to assess the time of onset of nutritional support and mortality.
Merhar et al., 2015 [13]	USA	Hospital	24	13	15 M 9 F	Uninformed	Enteral	A study evaluated the use of protein supplementation in children with CP for 12 months to maintain adequate weight gain. Children were weighed every 2 weeks and had adjusted doses (4 g/kg/day). Tolerance, bicarbonate levels, blood urea were also evaluated.
Miyazawa et al., 2008 [11]	Japan	Hospital	18	9	16 M 2 F	11.7 ± 4.4 years	Enteral nasogastric	A study evaluated the use of high amounts of pectin and low amounts of pectin to control GERD in children with CP. Assessment number of vomiting, residual gastric volume, esophageal pH.
Savage et al., 2015 [15]	Australia	Hospital	13	7	8 M 5 F	7.2 years [CI 2.4–15.4 years]	Enteral via gastrostomy and nasogastric	A study sought to determine whether whey-based versus casein-based enteral formulas reduce reflux and accelerate gastric emptying in children with severe CP enteral feeding. He also evaluated the effect of these formulas on symptoms of low food tolerance such as choking, regurgitation, irritability, pain, and constipation.

**Table 2:** Main outcomes.

Author/Year	Intervention/Dose	Evaluated Outcomes	Supplementation Characteristics	Main Results	Authors’ Conclusions
Andrew et al., 2017 [16]	The babies received docosahexaenoic acid (DHA), choline and uridine-5-monophosphate (UMP), 2 g/kg/day (maximum 24 g/day), supplied as a combination of 2 g, 3 g and 12 g sachets, for 2 years or while complying with the study protocol.	Neurodevelopment of neonates was assessed by the Bayley-III is a standardized measure of neurodevelopment suitable for children aged 1 to 42 months with cognitive, language, and motor domains.	Participants received daily treatment for 2 years. The intervention group received supplementation with 37.8 mg of docosahexaenoic acid, 7.8 mg of eicosapentaenoic acid, 4.4 mg of arachidonic acid, 1.8 mg of uridine monophosphate, 1.8 mg of cytidine monophosphate, 10.5 mg choline, 0.12 µg vitamin B12, 0.76 mg zinc, 15 µg iodine. The control group received supplementation with 0.4 mg of docosahexaenoic acid, 0.08 mg of eicosapentaenoic acid, 0.02 mg of arachidonic acid, 0 mg of uridine monophosphate, 0 mg of cytidine monophosphate, 1.38 mg of choline, 0.02µg vitamin B12, 0.06 mg of zinc, 0.6 µg of iodine. Babies received 2 g/kg/day (maximum 24 g/day), supplied as a combination of 2 g, 3 g, and 12 g sachets, for 2 years or in accordance with the study protocol. The supplement was mixed with the usual formula or expressed breast milk and with moist foods at weaning or delivered through a feeding tube.	The Cognitive composite score of the Bayley Scales of Infant and Toddler Development, Third Edition (CCS Bayley-III) treatment group was not significantly larger than the comparison group (mean 77.7 [SD 19.2] and 72.2 [SD 19.8] *p* = 0.18). The language scores of the treatment group, but not the motor scores, were not significantly higher than for the comparison groups.	No statistically significant differences were identified in the result of neurological development between the treatment and comparison groups.
Briassoulis et al., 2006 [17]	Enteral feeding supplemented with glutamine, arginine, antioxidants and omega-3 fatty acids	Nutritional and metabolic indices; survival, length of stay, ventilation time. The severity of the head injury was assessed by the Glasgow Coma Scale, and the severity of illness was assessed by the Pediatric Risk of Mortality (PRISM) score, the simplified Therapeutic Intervention Scoring System (TISS), and indices of organ failure. Multiple organ system failures were defined using the criteria by Wilkinson. Caloric intake was calculated daily from the patients’ flow sheets. In each group, were calculated the differences of caloric intake-PBMR, caloric intake–predicted energy expenditure, and dactual caloric or protein intake–initially estimated caloric or protein difference.	Of the 40 mechanically ventilated children with severe head trauma, 20 were in the intervention group using Stresson (N. V. NUTRICIA, Zoetermeer, The Netherlands), an enteral food supplemented with glutamine, arginine, antioxidants and omega-3 fatty acids and 20 were in the control group that used Tentrini (N. V. NUTRICIA), an enteral formula specifically modified for critically ill children. All food was delivered through a nasogastric tube, started within the first 12 hours of admission. The hourly amount was calculated according to the following protocol: energy consumption equal to 0.50%, 100%, 125%, 150%, and 150% of PBMR on days 1 to 5, respectively.	Only interleukin -8 levels were lower in the intervention group compared to the regular formula group on day 5 (23.6 1.5 vs. 35.5 4 pg/mL, *p* < 0.04). In multivariate regression analysis, interleukin −8 was also independently negatively correlated with immunonutrition (*p* < 0.04). The nitrogen balance became positive in 30.8% of patients in the regular formula group and in 69.2% of patients in the study group on day 5 (*p* < 0.05). Fewer gastric cultures were positive in the study group compared to the regular formula group (26.7% vs. 71.4%, *p* < 0.02). Hospital infections (15% vs. 25%), length of stay (16.7 vs. 12.2 days), time on mechanical ventilation (11 vs. 8 days), and survival (80% vs. 95%) did not differ between groups.	Although immunonutrition can decrease interleukin-8 and gastric colonization in children with severe TBI, it may not be associated with additional advantage over that demonstrated by regular early enteral nutrition.
Dabydeen et al., 2015 [14]	120% of the EAR for energy in the first 6 months and 101% in the 6 months after. Protein/energy ratio was 2.5 g/100 kcal to 3.6 g/100 kcal	Brain and body growth rate. Measurements were made at baseline (term) and final measurements at 12 months; intermediate measurements were also made to provide information on the pattern of growth in the first 12 months. Head circumference and weight were, therefore, also measured at 3 monthly intervals, corticospinal tract axonal diameter was also estimated at 4 and 8 months, and the length was also measured at 6 months. SD (z) scores for anthropometric measures, derived from the British 1990 growth reference, which was revised in September 1996, were used so that age and gender data could be combined. Weight was measured to the nearest 10 g, with the child unclothed, by using a portable digital electronic scale. The length was measured using a horizontal stadiometer accurate to 1 cm. For both weight and length, 3 measurements were made, and the mean was calcu- lated.	All children were fed orally. For both groups, the goal of their protein/energy ratio was 2.5 g/420 kJ (100 kcal) to 3.6 g/420 kJ (100 kcal).	The study ended in the first analysis, when the 16 subjects completed the protocol, since the predetermined stop criterion of 1 SD difference in occipitofrontal circumference at 12 months of age corrected in those who received the high energy and protein diet was demonstrated. Axonal diameters in the corticospinal tract (*p* = 0.001), length (*p* = 0.04), and weight (*p* = 0.05) also increased significantly.	The results show that babies with significant perinatal brain damage increased nutritional needs in the first postnatal year and suggest that decreased postnatal brain growth may exacerbate their deficiency.
Meinert et al., 2018 [18]	Children were divided into 4 groups according to the time to start nutritional support and hypothermia or normothermia. Group 1—without nutritional support during the study period; Group 2—nutritional support started <48 hours after the injury; Group 3—nutritional support started 48 h–<72 h after the injury; Group 4—nutritional support started 72 h–168 h after injury.	Glasgow Mortality and Outcome Scale. Outcome measures addressed both mortality and functional outcome (Glasgow Outcome Scale score extended for Pediatrics [GOS-E Peds] at 6 months and 12 months after injury. For this analysis, GOS-E Peds was stratified into three groups; favorable outcome (GOS-E Peds = 1–4); unfavorable outcome (GOS-E Peds = 5–7), and dead (GOS-E Peds = 8).	The characteristics of the diet are not described in the study, only the period of the beginning of the diet and its outcomes were evaluated.	The beginning of nutrition before 72 h was associated with survival between Group 1 and Groups 2 and 3 were statistically significant (*p* = 0.001 and 0.006, respectively), while Groups 1 and 4 tended to be different (*p* = 0, 06). Likewise, the time to start nutrition was associated with GOS-E Peds at 6 m and 12 m (*p* = 0.03 and 0.04). At both time points, Group 1 had lower GOS-E Peds scores compared to Groups 2 and 3 (6 m: *p* = 0.007 and 0.02, respectively; 12 m: *p* = 0.005 and *p* = 0, 03, respectively), indicating that the onset of earlier nutrition was associated with improved GOS-E Peds compared to the group that did not receive nutrition during the study period. The time to start nutrition was associated with mortality (*p* = 0.05) with Group 1 having a 22 times greater chance of mortality when compared to Group 2.	The beginning of nutritional support before 72 hours after TBI was associated with decreased mortality and a favorable outcome in this analysis.
Merhar et al., 2015 [13]	Use of 4 g/kg/d or a maximum of 30 g of protein per day	weight gain, growth, and tolerance. Growth parameters were followed as per usual clinical practice while infants were inpatients in the NICU, with head circumference and length measured once a week and weight measured daily by the patient’s bedside nurse. After discharge, weight, length, and head circumference were measured by an examiner blinded to the patient’s study group at 3 months (±2 weeks) of age. Measurements were done on the same scale and length board for all participants in our NICU follow-up clinic.	A high-protein diet was offered to members of the high-protein group that achieved complete enteral nutrition (of at least 130 mL/kg/d), protein was added to the diet to bring them to a target of 4 g/kg/d of protein, with a maximum of 30 g of protein per day. The protein supplement used was Beneprotein Instant Protein Powder (Nestlé Health Care Nutrition, Florham Park, NJ), the protein source is a whey protein isolate containing 6 g of protein and 25 kcal per 7 g of powder.	Whey protein powder was well tolerated by 9 of the 13 children in the high-dose protein group, and no adverse events related to the supplement were seen. The protein group had higher serum urea nitrogen levels at 10 (*p* < 0.0001) and 30 (*p* = 0.0001) days after the start of the study, but no difference in bicarbonate levels at any point in time. Babies in the protein group maintained their weight z score from birth to 3 months of age, while babies in the standard group had a significant decrease (*p* = 0.03) in their weight z score over the same period of time.	Protein supplementation maintained the growth rate in infants with brain damage. Whey protein supplementation can potentially contribute to tissue growth and repair after brain injury. There was no difference between the two groups in birth weight at birth, however at 3 months of age the control group had a significant decrease in weight, while the protein group maintained a weight within the recommended values.
Miyazawa et al., 2008 [11]	Pectin supplementation in two dosages: 2:1 high pectin (Group A) and 3:1 low pectin (Group B)	Gastroesophageal reflux (GER).In the first part of this study, esophageal pH monitoring was performed over 48 hours for each subject. A single crystal antimony multi-use pH catheter with two channels [the end of the catheter, and 7 cm (5 cm for two patients under 6 years of age) above the end] was placed and connected to a portable digital data recorder. In the second part of the study, to elucidate the clinical effects of pectin liquid on GERD symptoms, four patients in group A were fed with a high-pectin diet for 4 weeks, followed by a non-pectin diet for 4 weeks. Five other patients were fed in reverse order. Nine patients in group B were fed with a low-pectin or non-pectin diet.	They received the enteral formula through a nasogastric tube. Group A patients were fed the enteral formula, including a high concentration of pectin liquid [enteral formula: pectin liquid = 2:1. Group B received a low pectin diet [enteric formula: pectin liquid = 3:1)] or non-pectin diet. The composition of the enteric formula per 100 mL was 100 kcal, 4.5 g protein, 16 g carbohydrate, and 2.7 g lipid. The composition of the pectin liquid per 100 mL was 9 kcal, 0.2 g protein, 0.6 g carbohydrate, 0.1 g lipid, and 76 mg sodium.	The mean value for pH% of time <4 in the lower and upper esophagus was significantly reduced with a diet rich in pectin [9.2% (6.2–22.6) vs. 5.0% (3.1–13.1); *p* < 0.01, 3.8% (2.9–11.2) vs. 1.6%(0.9–8.9); *p* < 0.01 (interquartile range), without pectin and high pectin, respectively. The number of reflux episodes per day and the duration of the longest reflux decreased significantly with a high pectin, but not with a low pectin diet. The average number of vomiting episodes decreased significantly with a high dietary perspective [2.5/week (1.0–5.0) vs. 1.0 (1.0–1.5), *p* < 0.05]. The mean cough score was significantly decreased at both pectin concentrations [8.5/week (1.0–11.5) vs. 2.0/week (0.0–3.0), fed with high pectin; 7.0/week (1.0–14.5) vs. 1.0/w (0.0–5.0), fed a low pectin diet, *p* < 0.05].	He observed that liquid pectin can help and improve the reduction of vomiting episodes, respiratory symptoms, and gastroesophageal reflux in children with CP, and can be considered an alternative therapy for gastroesophageal reflux disease in these patients.
Savage et al., 2015 [15]	Comparison of the use of three protein components. Casein-based enteric formula [82% casein, 18% whey] with formula 50% whey, 50% casein (50% WWP), or 100% partially hydrolyzed whey protein (100% WPHP)	Reflux and gastrointestinal symptoms. On day 6 of each week, GE rate was measured using the 13C-Na-octanoate breath test using 13C-labeled Na-octanoate (50 mg, 99% enrichment). Patients were fasted overnight and then given a 200-mL bolus of either the casein or whey formula containing 100 mg 13C-Na-octanoate for breath test measurement of liquid GE. Breath samples were taken using a small flexible tube connected to a syringe, which was held in close proximity to the patient’s mouth or nose as he or she breathed out. Samples were taken before the bolus and afterward at 5-min intervals until 30-min and 15-min intervals until 4 h. Patients remained recumbent in their wheelchair, in a pram, or on a hospital bed for the 4-h study period. Breath samples were analyzed for 13CO2 content using an isotope ratio mass spectrometer, and the 13CO2 excretion rate curves were used to calculate the gastric half-emptying time (GE t1/2) using an established nonlinear regression model. Age-related reference ranges20 for liquid GE t1/2 were then used to compare GE with various formulas.	The patients acted as their own controls and the children were divided into three groups where they received for a week: standard casein-based enteric formula (Pediasure [82% casein, 18% whey); Abbott Australasia, NSW, Australia) or 50% whey, 50% casein Formula (50% WWP) (Nutren Junior; Nestlé Clinical Nutrition, Vevey, Switzerland) or 100% partially hydrolyzed whey protein (100% WPHP) formula (Peptamen Junior; Nestlé Clinical Nutrition).	Whey formulas emptied significantly faster than casein (*p* = 0.033). The reflux parameters have not been changed. The symptoms of gastrointestinal discomfort were lower (*p* = 0.035) and lower pain score (*p* = 0.014) in children who received 50% WWP compared to those with 100% WPHP.	In children with severe CP with gastrostomy, gastric emptying of the whey-based enteric formula is significantly faster than casein. The acceleration in gastric emptying does not change the frequency of gastroesophageal reflux, and there seems to be no effect of whey versus casein in reducing episodes of acid, non-acid, and total reflux. The results indicate that the selection of enteral formula may be particularly important for children with severe CP and delayed gastric emptying.
Staiano et al., 2000 [12]	Supplementation of 100 mg/kg of glucomannan 2× daily or placebo (both 500 mg capsules)	Chronic constipation in children with severe brain injury. Weekly defecation frequency, total gastrointestinal transit time, colonic segmental transit time, and anorectal manometry were evaluated in all patients, before and after treatment. In brief, total gastrointestinal transit time was assessed by means of 20 polyethylene radiopaque markers (5-mm diameter), swallowed with a milk breakfast. The feces from each subsequent bowel movement were collected and radiographed to evaluate the presence and number of radiopaque markers until excretion of at least 80% of the markers was documented. Colonic segmental transit time was evaluated by using a plain abdominal radiograph performed after 24 h from the last ingestion of 3 sets of 20 distinctive markers (ring, cylinder, and linear) taken on 3 consecutive days. The 3 types of markers were always ingested in the same sequence.	The patients were divided into treatment with glucomannan 100 mg/kg twice daily or placebo. An oral dose was administered by mixing the contents of a 500 mg capsule with 100 mL of water; the result was a solution containing glucomannan in the final concentration of 5 mg/ml,	The group using glucomannan significantly increased (*p* < 0.01) the frequency of stools. Regarding the use of laxative or suppository, the use was significantly reduced (*p* < 0.01) by the glucomannan group. Clinical scores for stool consistency were significantly improved and episodes of painful defecation per week were significantly reduced by the use of glucomannan (*p* < 0.01).	An increase in the frequency of evacuation was observed for the intervention group. Laxative use reduced in the intervention group. There was also an improvement in the consistency of evacuation with reduced pain when defecating in the intervention group compared to the control group. In children with neurological problems, glucomannan had a beneficial effect only on bowel habits and not on gastrointestinal transit time.

## Data Availability

The primary manuscripts included are available at Pumed. Tables and analyzes performed can be found by sending an email to the author.

## References

[1] Levin H.S., Hanten G. (2005). Executive Functions After Traumatic Brain Injury in Children. Pediatr. Neurol..

[2] Thurman D.J. (2016). The epidemiology of traumatic brain injury in children and youths: A review of research since 1990. J. Child Neurol..

[3] Lumba-Brown A., Yeates K.O., Sarmiento K., Breiding M.J., Haegerich T.M., Gioia G.A., Turner M., Benzel E.C., Suskauer S.J., Giza C.C. (2018). Centers for Disease Control and Prevention Guideline on the Diagnosis and Management of Mild Traumatic Brain Injury Among Children. JAMA Pediatr..

[4] Bass N. (1999). Cerebral palsy and neurodegenerative disease. Curr. Opin. Pediatr..

[5] Rotta N.T. (2002). Cerebral palsy, new therapeutic possibilities. J. Pediatr..

[6] Korzeniewski S.J., Slaughter J., Lenski M., Haak P., Paneth N. (2018). The complex aetiology of cerebral palsy. Nat. Rev. Neurol..

[7] Stavsky M., Mor O., Mastrolia S.A., Greenbaum S., Than N.G., Erez O. (2017). Cerebral Palsy—Trends in Epidemiology and Recent Development in Prenatal Mechanisms of Disease, Treatment, and Prevention. Front. Pediatr..

[8] Jahan I., Muhit M., Karim T., Smithers-Sheedy H., Novak I., Jones C., Badawi N., Khandaker G. (2019). What makes children with cerebral palsy vulnerable to malnutrition? Findings from the Bangladesh cerebral palsy register (BCPR). Disabil. Rehabilit..

[9] Arvedson J.C. (2013). Feeding children with cerebral palsy and swallowing difficulties. Eur. J. Clin. Nutr..

[10] Sullivan P.B. (2013). Nutrition and growth in children with cerebral palsy: Setting the scene. Eur. J. Clin. Nutr..

[11] Miyazawa R., Tomomasa T., Kaneko H., Arakawa H., Shimizu N., Morikawa A. (2008). Effects of pectin liquid on gastroesoph-ageal reflux disease in children with cerebral palsy. BMC Gastroenterol..

[12] Staiano A., Simeone D., Del Giudice E., Miele E., Tozzi A., Toraldo C. (2000). Effect of the dietary fiber glucomannan on chronic constipation in neurologically impaired children. J. Pediatr..

[13] Merhar S.L., Meinzen-Derr J., Sprague J., Wessel J.J., Leugers S., Painter J., Valentine C.J. (2015). Safety and Tolerability of Enteral Protein Supplementation for Infants with Brain Injury. Nutr. Clin. Pr..

[14] Dabydeen L., Thomas J.E., Aston T.J., Hartley H., Sinha S.K., Eyre J.A. (2008). High-Energy and -Protein Diet Increases Brain and Corticospinal Tract Growth in Term and Preterm Infants After Perinatal Brain Injury. Pediatry.

[15] Savage K., Kritas S., Schwarzer A., Davidson G., Omari T. (2012). Whey- vs Casein-Based Enteral Formula and Gastrointestinal Function in Children with Cerebral Palsy. J. Parenter. Enter. Nutr..

[16] Andrew M.J., Parr J.R., Montague-Johnson C., Laler K., Qi C., Baker B., Sullivan P.B. (2017). Nutritional intervention and neurodevelopmental outcome in infants with suspected cerebral palsy: The Dolphin infant double-blind randomized controlled trial. Dev. Med. Child Neurol..

[17] Briassoulis G., Filippou O., Kanariou M., Papassotiriou I., Hatzis T. (2006). Temporal nutritional and inflammatory changes in children with severe head injury fed a regular or an immune-enhancing diet: A randomized, controlled trial. Pediatr. Crit. Care Med..

[18] Meinert E., Bell M.J., Buttram S., Kochanek P.M., Balasubramani G.K., Wisniewski S.R., Adelson P.D. (2018). Initiating nutritional support before 72 hours is associated with favorable outcome after severe traumatic brain injury in children: A secondary analysis of a randomized, controlled trial of therapeutic hypothermia. Pediatr. Crit. Care Med..

[19] Chiang Y.-H., Chao D.-P., Chu S.-F., Lin H.-W., Huang S.-Y., Yeh Y.-S., Lui T.-N., Binns C.W., Chiu W.-T. (2012). Early Enteral Nutrition and Clinical Outcomes of Severe Traumatic Brain Injury Patients in Acute Stage: A Multi-Center Cohort Study. J. Neurotrauma.

[20] Balakrishnan B., Flynn-O’Brien K.T., Simpson P.M., Dasgupta M., Hanson S.J. (2019). Enteral nutrition initiation in children ad-mitted to pediatric intensive care units after traumatic brain injury. Neurocrit. Care.

[21] Taha A.A., Badr L., Westlake C., Dee V., Mudit M., Tiras K.L. (2011). Effect of Early Nutritional Support on Intensive Care Unit Length of Stay and Neurological Status at Discharge in Children with Severe Traumatic Brain Injury. J. Neurosci. Nurs..

[22] Karim T., Jahan I., Dossetor R., Giang N.T.H., Van Anh N.T., Dung T.Q., Chau C.M., Van Bang N., Badawi N., Khandaker G. (2019). Nutritional Status of Children with Cerebral Palsy—Findings from Prospective Hos-pital-Based Surveillance in Vietnam Indicate a Need for Action. Nutrients.

[23] Schoendorfer N., Tinggi U., Sharp N., Boyd R., Vitetta L., Davies P.S. (2011). Micronutrient intakes in enterally and orally fed children with severe cerebral palsy. e-SPEN Eur. e-J. Clin. Nutr. Metab..

[24] Brun A.C., Størdal K., Johannesdottir G.B., Bentsen B.S., Medhus A.W. (2012). The effect of protein composition in liquid meals on gastric emptying rate in children with cerebral palsy. Clin. Nutr..

[25] García-Contreras A.A., Vásquez-Garibay E.M., Romero-Velarde E., Ibarra-Gutiérrez A.I., Troyo-Sanromán R., Sandoval-Montes I.E. (2014). Intensive nutritional support improves the nutritional status and body composition in severely mal-nourished children with cerebral palsy. Nutr. Hosp..

